# Technology Guided Assessment for Urinary Tract Infection: Creating a Common Interprofessional Language

**DOI:** 10.1093/geroni/igab046.3202

**Published:** 2021-12-17

**Authors:** Donna Owen, Alyce Ashcraft, Kyle Johnson, Huaxin Song, John Culberson

**Affiliations:** 1 Texas Tech University Health Sciences Center, Houston, Texas, United States; 2 Texas Tech University Health Sciences Center, Lubbock, Texas, United States

## Abstract

The Shared Meaning Model (SMM) is a grounded theory, derived in a previous study. This model demonstrates pathways for communication between nurse and primary care providers (PCPs) in the nursing home (NH), In this study we used the SMM for feasibility testing of a clinical decision support app (CDS app) using a descriptive, structured observational design. This study also provided a forum for initial testing of the SMM. The CDS app algorithm provided a common language to assess a resident with the goal of sharing this information with a PCP. The CDS app guided licensed vocational nurses (LVNs) (N=10) in assessing a standardized nursing home resident in a simulation setting experiencing symptoms of a potential urinary tract infection (UTI). Interviews with LVNs provided details of CDS app usability and concerns about using the CDS app with NH residents. Videos recorded LVNs interacting with the resident while using the CDS app on an iPad®. Time-stamps logged duration of the assessment. Bookmarked segments were used for discussion in LVN interviews. Videos were coded for eye contact, conversation, and touch between LVN and resident and documented personalized interactions. Findings indicated areas (lab values, drug names) for changes to language in the algorithm. In less than 12 minutes the CDS app enabled LVNs to collect information based on language used by PCPs to make decisions about the presence of a UTI. Relationships between initial constructs in the SMM were supported. This CDS app holds promise for building a common language to enhance interprofessional communication.

